# Diagnostic value of serum neuroactive substances in the acute exacerbation of chronic obstructive pulmonary disease complicated with depression

**DOI:** 10.1515/biol-2022-0693

**Published:** 2023-08-29

**Authors:** Wei Duan, Mengyu Cheng

**Affiliations:** Department of Respiratory and Critical Care Medicine, Shanxi Bethune Hospital, Shanxi Academy of Medical Sciences, Tongji Shanxi Hospital, Third Hospital of Shanxi Medical University, Taiyuan, 030032, China; Department of Respiratory and Critical Care Medicine, Tongji Hospital, Tongji Medical College, Huazhong University of Science and Technology, Wuhan, 430030, China

**Keywords:** chronic obstructive pulmonary disease, acute exacerbation, depression, γ-aminobutyric acid, glycine, nitric oxide

## Abstract

We aimed to investigate the potential diagnostic value of five serum neuroactive substances in patients with acute exacerbation of chronic obstructive pulmonary disease (AECOPD) complicated with depression. A total of 103 patients with AECOPD were enrolled between August 2020 and August 2021. All patients were assessed using a self-rating depression scale and divided into AECOPD with or without depression groups. Baseline data and serum neuroactive substance levels were compared between the two groups. Logistic regression was used to identify the risk factors. The diagnostic performance of neuroactive substances was evaluated using receiver operating characteristic (ROC) curves. Patients with AECOPD complicated with depression exhibited higher partial pressure of CO_2_ values and higher chronic obstructive pulmonary disease assessment test (CAT) scores. An elevated proportion of patients with more than two acute exacerbations (AEs) in the previous year was observed in this patient group (all *P* < 0.001). The CAT score and number of AEs during the previous year were identified as independent risk factors for AECOPD complicated with depression. No significant differences were observed in the levels of aspartic acid and glutamate between the two groups (*P* > 0.05). Serum γ-aminobutyric acid (GABA) and glycine (Gly) levels were decreased. In contrast, serum nitric oxide (NO) levels were increased in the AECOPD complicated with the depression group (*P* < 0.05). Serum GABA and Gly levels exhibited a negative correlation, and NO levels positively correlated with the number of AEs in the previous year and the CAT score. The area under the ROC curve values for GABA, Gly, and NO were 0.755, 0.695, and 0.724, respectively. Serum GABA exhibited a sensitivity of 85.1% and a specificity of 58.9%, below the cut-off value of 4855.98 nmol/L. Serum GABA, Gly, and NO may represent potential biomarkers for AECOPD complicated with depression.

## Graphical abstract

**Figure:**
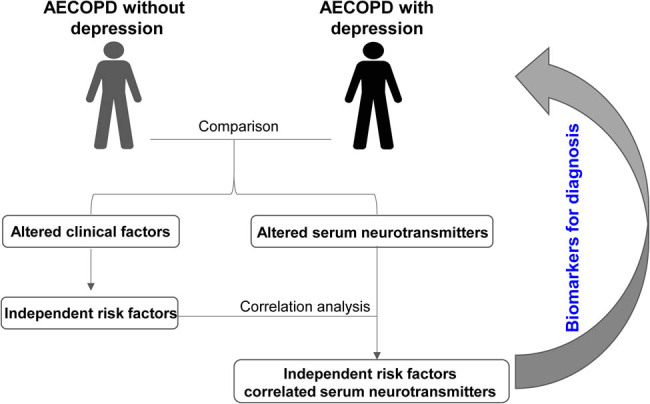

## 1 Introduction

Chronic obstructive pulmonary disease (COPD), caused by exposure to inhaled noxious particles, mainly those present in tobacco smoke and gaseous pollutants, is the most common chronic respiratory disease and is considered a major cause of increased morbidity, mortality, and healthcare burden throughout the world [1]). A recent systematic review and modeling study revealed that when using the Global Initiative for Chronic Obstructive Lung Disease (GOLD) definition, the incidence of COPD worldwide was 10.3% among individuals aged 30–79 in 2019 and accounted for approximately 391.9 million cases that occurred predominantly in low- and middle-income countries [2]. COPD is associated with multiple comorbidities such as osteoporosis, cardiovascular disease, and depression [3,4], which can negatively affect disease prognosis.

Depression is one of the most common comorbidities of COPD. The prevalence of depression has been reported at 7–80% in outpatients with COPD and 10–42% in the stable stage of COPD [5]. For example, in a cross-sectional observational study of 530 stable patients with COPD, 21.5% of patients suffered depression [6]. Depression is associated with decreased treatment compliance, a poor prognosis, high mortality, frequent hospitalizations, and poor quality of life [7]. Current evidence suggests a bidirectional association between COPD and clinical depression [8]. COPD increases the risk of developing depression. A large UK population-based cohort study indicated a depression incidence rate of 11.4 per 1,000 person-years in patients with COPD and 5.7 per 1,000 person-years in COPD-free populations. The patients with COPD exhibited a 41% higher risk of depression than those without COPD after adjustment [9]. In contrast, comorbid depression consistently increases the risk of exacerbation and mortality [8].

Nevertheless, the diagnosis of comorbid depression by respiratory clinicians is challenging and inadequate due to its overlapping symptoms with COPD. Less than one-third of the patients receive timely diagnosis and effective treatment [10]. Evidence indicates that gaseous neurotransmitters such as nitric oxide (NO) and amino acid neurotransmitters such as glutamate (Glu), γ-aminobutyric acid (GABA), aspartic acid (Asp), and glycine (Gly) play important roles in the pathogenesis of depression [11,12]. Changes in neurotransmitter levels have been reported in the cerebrospinal fluid, different brain regions, urine, and blood of patients with depressive disorder. Thus, they can be used as biomarkers for evaluating nervous system activities [13,14]. Additionally, these neurotransmitters are involved in COPD. For example, NO was determined to be a crucial effector in airflow obstruction and the development of COPD [15]. Multiple neurotransmitters and receptors, including GABA, Glu, and Gly, have been reported to be expressed in bronchial epithelial cells and interact with each other in a manner similar to that of neuron-like interactions to regulate physiological functions [16]. For example, GABA secreted by lung neuroendocrine and airway epithelial cells plays significant roles in regulating airway mucus secretion and the development of pulmonary hypertension [16,17]. However, only a few studies have investigated the associations of these neuroactive substances with COPD and depression.

Acute exacerbation of COPD (AECOPD) is characterized by the acute worsening of respiratory symptoms [18]. AECOPD and depression frequently co-occur. In a prospective observational study based on 288 patients with AECOPD, depression was confirmed in 41.7% of patients, and probable depression was diagnosed in 67.7% [19]. Therefore, depression should be systematically screened in patients with AECOPD, and timely and personalized therapeutic interventions should be provided. In this study, we aimed to investigate the potential value of neuroactive substances as biomarkers for screening patients with AECOPD complicated with depression. Our findings can aid in screening, diagnosing, and treating AECOPD complicated with depression.

## 2 Methods

### 2.1 Study design

Patients diagnosed with AECOPD (*n* = 103) in the Department of Respiratory and Critical Care Medicine at the Third Hospital of Shanxi Medical University from August 2020 to August 2021 were recruited for this study. Depression was assessed in all patients, and they were assigned to the AECOPD without depression and AECOPD with depression groups. All enrolled patients signed informed consent forms before they participated in this study. This study was approved by the Ethics Committee of the Third Hospital of Shanxi Medical University (Approval No. YXLL-2019-073) and complied with the Declaration of Helsinki.


**Informed consent:** Informed consent has been obtained from all individuals included in this study.
**Ethical approval:** The research related to human use has been complied with all the relevant national regulations, institutional policies and in accordance with the tenets of the Helsinki Declaration, and has been approved by Ethics Committee of the Third Hospital of Shanxi Medical University (Approval No. YXLL-2019-073).

### 2.2 Inclusion and exclusion criteria

Inclusion criteria were as follows: (1) COPD was diagnosed based on the 2020 GOLD diagnostic criteria for COPD [20]; (2) AECOPD was determined according to the definition of an acute worsening of respiratory symptoms (including an increased degree of dyspnea and cough, purulent sputum, or increases in sputum) that resulted in the need for additional treatment; (3) patients greater than 40 years of age who possessed normal cognitive and language skills and were capable of barrier-free communications; and (4) patients with reimbursable medical expenses and fixed reimbursement rates for medical expenses according to the payment methods. Exclusion criteria were as follows: (1) patients with chronic respiratory diseases requiring treatment and intervention such as active pulmonary tuberculosis, pulmonary thromboembolism, interstitial pulmonary disease, chronic thromboembolic pulmonary hypertension, and others; (2) patients with extensive pleural effusion and pneumothorax and patients who cannot tolerate a 6-min walk; (3) patients who were diagnosed with mental illnesses such as anxiety, depression, and bipolar disorder prior to COPD diagnosis; (4) patients with severe cardiovascular disease and severe hepatic and renal insufficiency; (5) patients who had sequelae of cerebral infarction or cerebral hemorrhage and patients who had neuromuscular diseases; and (6) patients with solid tumors or hematological malignancy.

### 2.3 Data collection

Demographic and clinical pathological data, including age, sex, body mass index (BMI), number of pack-years of cigarette smoking, partial pressure of oxygen (PaO_2_), carbon dioxide (PaCO_2_), COPD assessment test (CAT) scores, pulmonary function, GOLD stages, number of acute exacerbations (AEs) in the previous year, and laboratory results within 24 h after hospitalization, were collected. The number of pack-years of cigarette smoking was defined as the number of packs (20 cigarettes) smoked per day multiplied by the total number of years of smoking [21]. Disease severity was categorized according to the GOLD staging criteria (GOLD stage I, II, III, or IV) that were determined depending on the post-bronchodilator forced expiratory volume in 1 s (FEV1)/forced vital capacity (FVC) [22]. The CAT score is a simple and reliable measure developed to evaluate the symptoms and impact of COPD on health status, and it involves eight items with a total score ranging from 0 to 40 [23,24].

### 2.4 Arterial blood gas and pulmonary function tests

Arterial blood (2 mL) was collected within half an hour after admission for use in arterial blood gas analysis on an automated blood gas analyzer (GEM-3500; USA), and PaO_2_, PaCO_2_, and oxygen absorption concentrations were recorded. The forced expiratory volume in 1 s as a percentage of predicted value (FEV1%pred) and FEV1/FVC were measured using the master-Screen pulmonary function test system (Jaeger Co., Germany).

### 2.5 Laboratory tests

Venous blood (5 mL) was collected on an empty stomach within 24 h after hospitalization, and supernatants were collected after centrifuging at 3,000 rpm for 20 min and subsequently utilized for determining the levels of NO, Glu, Asp, Gly, and GABA. Briefly, NO levels were determined using biochemical methods, and the levels of Glu, Asp, Gly, and GABA were determined using corresponding enzyme-linked immunosorbent assay kits (Shanxi Boao Xinyuan Biotechnology Co., LTD) according to the manufacturer’s protocol.

### 2.6 Self-rating depression scale (SDS)

The SDS is a widely used self-reporting measure designed based on the diagnostic criteria for depression that includes 20 items involving psychological and physiological symptoms, where 10 items express negative experiences and 10 items express positive experiences. Depression in all patients was assessed by rating each item according to how they felt within the past week using a 4-point scale. The score for each of the 20 items was added to obtain a raw score multiplied by 1.25 to generate a standard score. A standard score of 53 indicates clinically significant depression symptoms and includes three levels of severity: mild (standard score ranging from 53 to 62), moderate (standard score ranging from 63 to 72), and severe (standard score more than 72).

### 2.7 Statistical analysis

All statistical analyses were conducted using SPSS 25.0 (IBM, Armonk, New York). Measurement data subjected to normal distribution are represented as the mean ± standard deviation and were compared using independent sample *t*-tests. Measurement data that did not conform to the normal distribution are represented as the median (interquartile spacing) and were compared using the Wilcoxon rank-sum test. Counting data are provided as frequency counts and composition ratios and were compared using Chi-square tests. Spearman’s or Pearson’s correlation was used to explore the correlations between neuroactive substances and pathological characteristics. Logistic regression was used to identify the risk factors for patients with AECOPD. The diagnostic performance of neuroactive substances was evaluated using receiver operating characteristic (ROC) curves. Statistical significance was set at *P* < 0.05.

## 3 Results

### 3.1 Clinical baseline characteristics of patients

A total of 103 patients with AECOPD were enrolled, and they were assigned into AECOPD without depression (*n* = 56) and AECOPD with depression (*n* = 47) groups after the depression assessment, according to SDS. Table 1 lists the baseline demographic and clinicopathological data for all patients. Sex (male proportion: 85.1 vs 87.5%, *P* = 0.724) and mean age (68.9 ± 9.5 vs 66.6 ± 9.2, *P* = 0.226) were not significantly different between the AECOPD with or without depression groups. Additionally, BMI (21.9 ± 3.8 vs 22.9 ± 3.3, *P* = 0.142) and the number of pack-years of cigarette smoking (38 [30–50] vs 35 [23.5–49.5], *P* = 0.396), two factors that were considered confounding factors, were not significantly different between the AECOPD with or without depression groups. These results indicated high comparability between the two groups.

**Table 1:** Comparison of demographics and clinical pathological data between the AECOPD group and the AECOPD with depression group

	AECOPD with depression (*n* = 47)	AECOPD without depression (*n* = 56)	*χ* ^2^/*t*/*Z*	*P* value
Sex			0.125	0.724
Males	40 (85.1%)	49 (87.5%)		
Females	7 (14.9%)	7 (12.5%)		
Age (years)	68.9 ± 9.5	66.6 ± 9.2	−1.218	0.226
BMI (kg/m^2^)	21.9 ± 3.8	22.9 ± 3.3	1.482	0.142
Numbers of pack-years of cigarette smoking	38 (30–50)	35 (23.5–49.5)	−0.849	0.396
PaO_2_ (mmHg)	70.42 ± 10.81	70.75 ± 7.97	0.173	0.863
PaCO_2_ (mmHg)	43.66 ± 6.56	40.32 ± 5.53	−2.809	0.006
FEV1%pred	57.46 ± 16.22	59.46 ± 16.96	0.607	0.545
CAT score	26.34 ± 5.34	16.98 ± 5.46	−8.754	0.000
GOLD stages			−0.449	0.654
Stage I	5 (10.60%)	7 (12.5%)		
Stage II	25 (53.2%)	30 (53.6%)		
Stage III	12 (25.5%)	14 (25.0%)		
Stage IV	5 (10.6%)	5 (8.9%)		
Numbers of AEs in the previous year			−6.843	<0.001
0–1	5 (10.6%)	44 (78.6%)		
≥2	42 (89.4%)	12 (21.4%)		

AECOPD, acute exacerbation of chronic obstructive pulmonary disease; BMI, body mass index; PaO_2_, partial pressure of oxygen; PaCO_2_, partial pressure of carbon dioxide. FEV1%pred, forced expiratory volume in 1 s as percentage of predicted value; CAT, the COPD assessment test; GOLD, Global initiative for chronic obstructive lung disease.

Regarding clinical pathological characteristics, PaO_2_ (70.42 ± 10.81 vs 70.75 ± 7.97, *P* = 0.863), FEV1%pred (57.46 ± 16.22 vs 59.46 ± 16.96, *P* = 0.545), and GOLD stages (*P* = 0.654) did not exhibit statistically significant differences between the two groups. Patients in the AECOPD with depression group possessed higher PaCO_2_ than did those in the AECOPD without depression group (43.66 ± 6.56 vs 40.32 ± 5.53, *P* = 0.006). Elevated CAT scores (26.34 ± 5.34 vs 16.98 ± 5.46, *P* < 0.001) and proportion of patients with AEs in the previous year (>2 times, 42 [89.4%] vs 12 [21.4%], *P* < 0.001] in the AECOPD with depression group compared to these values in the AECOPD without depression group (Table 1) were observed.

### 3.2 Risk factors associated with AECOPD complicated with depression

Considering that patients in the AECOPD with depression group exhibited significantly increased PaCO_2_, CAT scores, and proportions of more than two AEs compared to those in the AECOPD without depression group, we further conducted logistic regression on these factors to identify the potential risk factors associated with AECOPD complicated with depression. As discussed in Table 2, the CAT score (1.271, 95% CI: 1.118–1.445, *P* < 0.001) and number of AEs in the previous year (12.069, 95% CI: 3.436–42.386, *P* < 0.001) were independent risk factors for AECOPD complicated with depression.

**Table 2:** Independent risk factors in logistic regression model

Variables	Coefficient	Standard error	*E*	*P*	95% CI
CAT score	0.24	0.065	1.271	0.000	1.118–1.445
Number of AEs in the previous year	2.491	0.641	12.069	0.000	3.436–42.386

CAT, the COPD assessment test. Logistic regression identified CAT score and number of AEs in the previous year as independent risk factors of AECOPD combined with depression.

### 3.3 Association of serum neuroactive substances with AECOPD complicated with depression

In addition to Glu and Asp, the other neuroactive substances exhibited statistically significant differences between the two groups (Table 3). Patients in the AECOPD with depression group possessed markedly decreased levels of serum GABA (4785.31 ± 73.35 vs 4860.62 ± 69.90, *P* < 0.001) and Gly (175.94 ± 7.22 vs 181.78 ± 9.48, *P* = 0.001) and markedly increased levels of serum NO (201.85 ± 9.48 vs 194.62 ± 6.71, *P* < 0.001) compared to levels in the patients in the AECOPD without depression group. Serum levels of GABA, Gly, and NO exhibited significant correlations with the two identified independent risk factors for AECOPD complicated with depression (Table 4). Serum levels of GABA and Gly were negatively correlated with both the CAT score (GABA, *r* = −0.347, *P* < 0.01; Gly, *r* = −0.264, *P* < 0.01) and the number of AEs in the previous year (GABA, *r* = −0.215, *P* < 0.05; Gly, *r* = −0.361, *P* < 0.01). The serum level of NO was positively correlated with the CAT score (*r* = 0.244, *P* < 0.05) and the number of AEs in the previous year (*r* = 0.238, *P* < 0.05). These findings suggest a close association of serum neuroactive substances (GABA, Gly, and NO) with AECOPD complicated with depression.

**Table 3:** Comparison of serum neuroactive substances between the AECOPD group and the AECOPD with depression group

	AECOPD with depression (*n* = 47)	AECOPD without depression (*n* = 56)	*t*	*P*
GABA (nmol/L)	4785.31 ± 73.35	4860.62 ± 69.90	5.325	0.000
Glu (μmol/L)	24.54 ± 1.62	24.27 ± 1.60	−0.865	0.389
Asp (μmol/L)	27.06 ± 1.78	27.69 ± 2.01	1.673	0.098
Gly (μmol/L)	175.94 ± 7.22	181.78 ± 9.48	3.46	0.001
NO (μmol/L)	201.85 ± 9.48	194.62 ± 6.71	−4.386	0.000

AECOPD, acute exacerbation of chronic obstructive pulmonary disease; GABA, gammaaminobutyric acid; Glu, glutamic acid; Asp, aspartic acid; Gly, glycine; NO, nitric oxide. Five serum neuroactive substances were compared between AECOPD petientis with or without depression, and three among which were significantly changed between groups in addition to aspartic acid (Asp) and glutamic acid (Glu).

**Table 4:** Correlations of serum neuroactive substances with independent risk factors

Variables	GABA	Gly	NO	Glu	Asp
CAT score	−0.347**	−0.264**	0.244*	−0.034	−0.052
Number of AEs in the previous year	−0.215*	−0.361**	0.238*	0.082	−0.187

CAT, the COPD assessment test; GABA, gammaaminobutyric acid; Glu, glutamic acid; Asp, aspartic acid; Gly, glycine; NO, nitric oxide. *P < 0.05; **P < 0.01. Correlations between levels of five serum neuroactive substances and two identified independent risk factors of AECOPD combined with depression were investigated, and significant correlations were found for three serum neuroactive substances in addition to aspartic acid (Asp) and glutamic acid (Glu).

### 3.4 Diagnostic value of serum neuroactive substances

**Figure 1:**
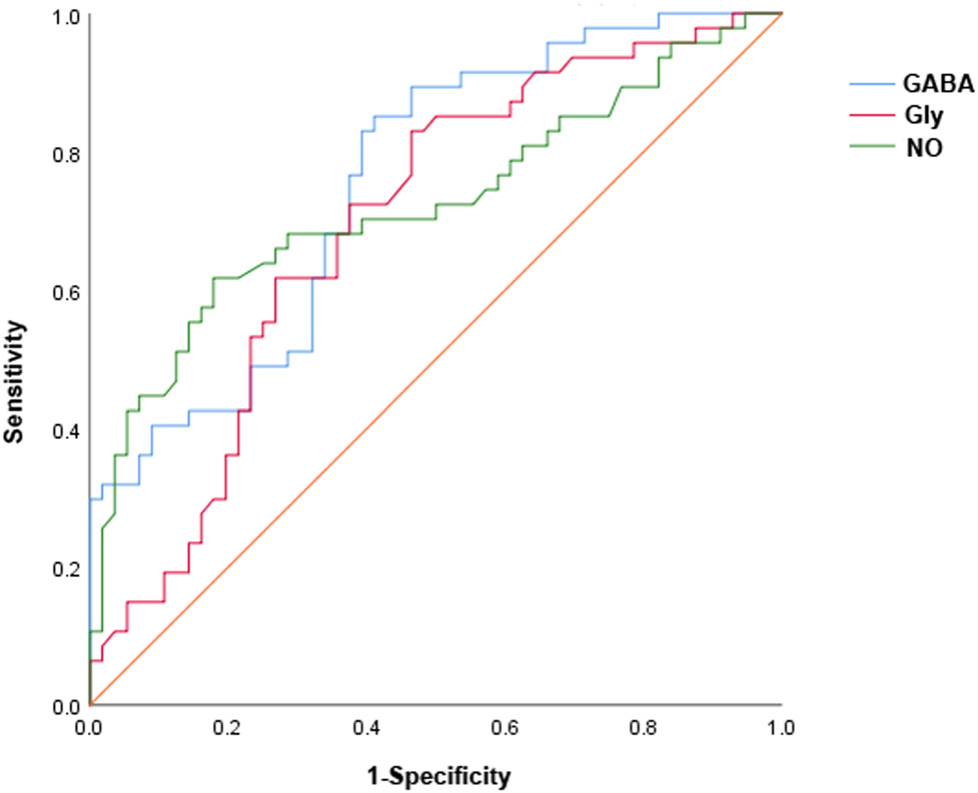
Diagnostic performance of γ-aminobutyric acid (GABA), Gly, and NO as evaluated using receiver operator characteristic curves.

Serum levels of GABA, Gly, and NO differed significantly between the two AECOPD groups. They were significantly correlated with the two independent risk factors. Based on this, their diagnostic value in AECOPD complicated with depression was further investigated (Figure 1; Table 5). The area under the ROC curve (AUC) of serum GABA for the diagnosis of AECOPD complicated with depression was 0.755 with a specificity of 58.9% and a sensitivity of 85.1% when serum GABA was <4,855.98 nmol/L. The AUC of serum Gly for the diagnosis of AECOPD complicated with depression was 0.695, with a specificity of 53.6% and a sensitivity of 83.0% when serum Gly was <180.01 μmol/L. The AUC of serum NO for the diagnosis of AECOPD complicated with depression was 0.724 with a specificity of 82.1% and a sensitivity of 61.7% when serum NO was >199.91 μmol/L. These results indicate that serum GABA possesses an optimal diagnostic value (largest AUC value and diagnostic sensitivity) compared to that of the other two neuroactive substances. However, the diagnostic specificity of serum GABA remains unsatisfactory.

**Table 5:** Diagnostic performance of GABA, Gly, and NO for AECOPD with depression

	AUC	Sensitivity (%)	Specificity (%)	95% CI	Cut-off value	*P*
GABA (nmol/L)	0.755	85.10	58.90	0.663–0.847	4855.98	<0.001
Gly (μmol/L)	0.695	83.00	53.60	0.593–0.797	180.01	0.001
NO (μmol/L)	0.724	61.70	82.10	0.622–0.826	199.91	<0.001

AUC, area under curves; GABA, gammaaminobutyric acid; Gly, glycine; NO, nitric oxide. Among five serum neuroactive substances, levels of only GABA, Gly, and NO were changed in AECOPD and significantly correlated with the independent risk factors of AECOPD combined with depression. Thus, the performance of serum GABA, Gly, and NO levels in diagnostic AECOPD was evaluated using receiver operator characteristic (ROC) curves. The area under ROC curves (AUC) was close to or greater than 0.7, indicating a good predictive performance.

## 4 Discussion

In this study, compared to those in patients with AECOPD without depression, we observed that serum levels of the neuroactive substances GABA and Gly were significantly decreased. The serum levels of NO were significantly increased in patients with AECOPD with depression symptoms. In addition, serum levels of GABA, Gly, and NO were closely correlated with the two independent risk factors (CAT score and number of AEs in the previous year) that were associated with AECOPD complicated with depression. Furthermore, ROC curves demonstrated the diagnostic value of GABA (AUC = 0.755), Gly (AUC = 0.695), and NO (AUC = 0.724) in the context of AECOPD complicated with depression. These findings indicated that serum GABA, Gly, and NO may be biomarkers to screen patients with AECOPD with depression.

Compared to the AECOPD without depression group, the AECOPD with depression group exhibited a high CAT score and a high proportion of more than two AEs in the previous year. This result was consistent with the results reported in a previous study [25]. In addition, these two factors were identified as independent risk factors. A previous study identified the CAT score as an independent risk factor for AECOPD with depression, and positive correlations between the CAT score and Hamilton Depression Rating Scale score (*r* ^2^ = 0.587) were observed [26]. Regarding AE numbers, it was observed that depression was related to an increased risk for exacerbations in COPD [27]. In a nationwide multicenter patient registry study, canonical correlation analysis revealed that 11% of the variance was shared between psychological symptoms (anxiety and depression) and four COPD outcome variables, including CAT, number of AEs, predicted FEV1%, and the modified medical research council dyspnea scale [28]. Further analysis revealed a positive correlation between depression and COPD outcomes, thus indicating that greater depression was associated with poorer CAT and more AEs [28]. This was consistent with the findings discussed in our study. In addition, depression was demonstrated to mediate the beneficial effect of social support on COPD outcomes. Higher social support was associated with better COPD outcomes in an adjusted cross-sectional analysis without depressive symptoms, while such associations were largely weakened when considering depressive symptoms. Depressive symptoms explained greater than 85% of the association between social support and measured COPD outcomes [29]. The GOLD stage was not correlated with any association between AECOPD and depression in this study (*P* = 0.654). A similar result (*P* = 0.093) was observed by Long et al. [26]. Most patients were in GOLD stages II–III, accounting for approximately 78% of all patients in this study. The association between AECOPD and depression and GOLD stages should be further investigated.

GABA is the most important inhibitory neurotransmitter in the mammalian central nervous system, and its dysfunction is involved in various mental and neurological diseases, such as depression and epilepsy [30,31]. Drugs that enhance GABAergic neurotransmission in the central nervous system have exhibited clinical benefits in treating mental and neurological diseases such as depression and epilepsy [31]. Positive depression status was reported to be related to lower levels of GABA-to-glutamate and GABA-to-glutamine ratios and higher levels of glutamate [32]. A study reported increased plasma GABA levels in patients with major depressive episodes [33]. GABA is expressed in bronchial epithelial cells, airway smooth muscle cells, and airway nerves. The GABAergic system in bronchial epithelial cells is excitatory rather than inhibitory, and its inhibition reduces mucus production [34]. Pulmonary neuroendocrine cells act through GABA to induce hyperplasia of goblet cells [35]. In addition, GABA was reported to reduce airway inflammation, and GABA in the epithelium exerted effects related to bronchial remodeling [36]. Gly is traditionally regarded as a non-essential amino acid and has been gradually recognized as being involved in tissue repair, oxidative stress, immunity, and metabolism in recent years [37,38]. Gly supplementation inhibited apoptosis and inflammation of alveolar cells and exerted a protective effect in pulmonary fibrosis [39,40]. Based on the observations described above, we speculated that the decreased serum GABA and Gly levels in patients with AECOPD with depression may be accompanied by increased airway inflammation.

NO, a potent vasoactive mediator, is generated from l-arginine by endothelial NO synthase. NO levels frequently change in patients with depression, and studies based on animal models have proposed that NO signals are targets of multiple antidepressants [41,42]. During the pathophysiology of major depression, NO signaling was observed to mediate inflammatory pathways [41]. Dysregulation of the endothelial NO pathway has been linked to airway inflammation in COPD [15]. Serum NO levels exhibited significant correlations with FEV_1_ and were independently associated with COPD [43]. A previous study reported inverse correlations between serum ADMA levels and NO levels in COPD [43]. In contrast, in another study, ADMA was reported to have positive correlations with blood neutrophil percentage and sputum neutrophil count [15]. Here, we observed that serum NO was increased in the AECOPD with depression group and that its expression was positively correlated with CAT score and AEs.

### 4.1 Strengths and limitations

This is the first study to investigate the associations between neuroactive substances and COPD complicated with depression. We demonstrated the potential predictive value of serum GABA, Gly, and NO levels regarding screening for AECOPD complicated with depression. However, this study does possess certain limitations. First, only patients with AECOPD were included, and patients with COPD in the stable stage, control individuals and/or stable depression without COPD were not analyzed. Second, depression in patients was evaluated using the SDS, which is a self-rating scale that reflects the emotional state of patients over a period of time. To assess depression more accurately, more than one scale (such as the Hamilton Depression Scale) should be used. Third, we preliminarily demonstrated the potential predictive value of GABA, Gly, and NO levels in AECOPD complicated with depression. Although the ROC curves indicated moderate predictive performance, the specificity of GABA and Gly was poor, and the sensitivity of NO was not high. Fourth, the sample size was small, and to ensure the reliability of the results, the sample size supporting this study design should be calculated in advance. Therefore, a well-designed study utilizing a larger sample size should further confirm the predictive value of GABA, Gly, and NO. The potential functions of GABA, Gly, and NO regarding the pathogenesis of AECOPD combined with depression should be explored.

## 5 Conclusion

In conclusion, our results revealed that serum GABA, Gly, and NO levels are significantly altered in patients with AECOPD complicated with depression compared to levels in those without depression and are closely correlated with two independent risk factors for AECOPD with depression: CAT score and number of AEs during the previous year. Therefore, serum GABA, Gly, and NO levels may provide useful biomarkers for AECOPD complicated with depression.
